# Hexamerization of Anti-SARS CoV IgG1 Antibodies Improves Neutralization Capacity

**DOI:** 10.3389/fimmu.2022.864775

**Published:** 2022-05-04

**Authors:** Kalyan Pande, Scott A. Hollingsworth, Miranda Sam, Qinshan Gao, Sujata Singh, Anasuya Saha, Karin Vroom, Xiaohong Shirley Ma, Tres Brazell, Dan Gorman, Shi-Juan Chen, Fahimeh Raoufi, Marc Bailly, David Grandy, Karthik Sathiyamoorthy, Lan Zhang, Rob Thompson, Alan C. Cheng, Laurence Fayadat-Dilman, Bernhard H. Geierstanger, Laura J. Kingsley

**Affiliations:** ^1^ Discovery Biologics, Merck & Co., Inc., South San Francisco, CA, United States; ^2^ Discovery Chemistry, Merck & Co., Inc., South San Francisco, CA, United States; ^3^ Infectious Disease and Vaccine Discovery, Merck & Co., Inc., West Point, PA, United States

**Keywords:** hexamer-enhancing IgG1 mutations, SARS-CoV, viral neutralization, complement, C1q binding

## Abstract

The SARS-CoV-2 pandemic and particularly the emerging variants have deepened the need for widely available therapeutic options. We have demonstrated that hexamer-enhancing mutations in the Fc region of anti-SARS-CoV IgG antibodies lead to a noticeable improvement in IC_50_ in both pseudo and live virus neutralization assay compared to parental molecules. We also show that hexamer-enhancing mutants improve C1q binding to target surface. To our knowledge, this is the first time this format has been explored for application in viral neutralization and the studies provide proof-of-concept for the use of hexamer-enhanced IgG1 molecules as potential anti-viral therapeutics.

## Introduction

Since the emergence of severe acute respiratory syndrome coronavirus 2 (SARS-CoV-2) in late 2019, the ongoing COVID-19 pandemic has caused significant, widespread, and irreparable harm worldwide. As of early March 2022, more than 6.05 million lives have been lost due to SARS-CoV-2 which itself has begun to mutate leading to multiple concerning variants spreading throughout the world (1–5). With over 220 million individual cases and counting, the need for potent therapeutics to fight this ongoing global threat cannot be understated.

SARS-CoV-2, like the related SARS-CoV-1 that emerged in the early 2000’s (6), enters the host cell through engagement by the trimeric S or spike protein displayed on the viral surface to a specifically targeted host membrane protein, angiotensin converting enzyme 2 (ACE2) (7–9). The binding of the trimeric spike to the host cell receptor *via* the receptor binding domain (RBD) triggers a cascade of events that result in the fusion of the viral and host membranes and ultimately host cell entry (10, 11). Given the importance of this interaction for virus entry and the prevalence of the spike on the viral surface, it is no surprise that the spike protein is among the most common targets of the host immune response (12).

Since 2003, numerous antibodies have been identified that neutralize SARS-CoV-1 and/or CoV-2 by binding to the RBD of the spike protein by either occupying the ACE2 binding site (13, 14) and preventing viral-host cell receptor complex formation or by binding elsewhere on the RBD and interfering with the effective host receptor engagement (15–17). In addition to those antibodies that target the RBD, there have also been antibodies identified that can instead target the N-Terminal Domain (NTD) (18, 19) and S2 regions (20, 21) of the spike protein.

Unfortunately, many antibodies against the coronavirus spike protein demonstrate binding, but little to no neutralization capacity (22, 23). Furthermore, in the context of the global pandemic where manufacturing can be a significant bottleneck (24), high potency antibodies that follow a traditional manufacturing path are most desirable. Recently, several publications have highlighted the potential for multivalent formats, including IgM and nanocages, to improve potency of anti-CoV-2 antibodies (25–28). This is a promising approach that has the potential to increase the potency of the existing pool of known antibodies. However, multivalent constructs often require more complex manufacturing and purification routes (29), can have more challenging pharmacokinetics, and may require specialized know-how (30). This is especially true for covalent multimers. In the context of the pandemic, this can be a significant hurdle to overcome.

Here we report improved neutralization in IgG1 molecules through the inclusion of two point mutations that enhance the native ability of IgG1 molecules to form a hexamer upon target engagement (31). Related mutations, specifically E430G, known to enhance the Fc-Fc interaction are currently being pursued in the clinic under the tradename Hexabody^®^ (31–33). Because multimerization occurs only upon target engagement, these hexamer- enhanced IgG1 molecules can be produced through a similar manufacturing process as typical IgG1 molecules.

In this study, we tested several published anti CoV-1 and CoV-2 IgG1 antibodies and demonstrated that enhancing hexamer formation using two Fc point- mutations can significantly improve viral neutralization in either pseudovirus or live virus setting.

## Materials and Methods

### Selection of Hexamer-Enhancing Mutants

There are a number of mutations reported that improve hexamer formation in IgG antibodies (32, 34). With a few notable exceptions (E345R for instance), the number of mutations roughly correlates with C1q binding, with triple mutants being the most prone to form hexamers, even in solution (34). Based on these findings we selected the dual mutant, E345R and E430G, herein referred to as the RG mutant, as proof of concept molecule. Given that the double mutant has been shown to have a slight propensity to multimerize in solution (34), we characterized the molecules by SEC and performed extra polishing to reduce any observed high molecular weight species. Notably, our assessment of the RG double mutant differs from that reported by Wang et al. (34) who observed multimerization in solution. This discrepancy could be due to the relatively low concentrations used or due to intrinsic differences between individual mAbs. While the RG double mutant may not be an ideal clinical candidate, it is thought to be adequate as a proof-of-concept molecule.

### Antibody Expression and Purification

Human IgG1 antibody heavy (HC) and light (LC) chains were cloned separately into our in-house mammalian expression vector with Lonza leader sequences for co-transfection. Plasmids for HC and LC were transiently transfected at 1:1 ratio into ExpiCHO expression system in suspension using serum-free defined media using the Max Titer Protocol (ThermoFisher). Cell culture supernatants were harvested after 7-days. High-throughput Protein-A based MAbSelect Sure affinity chromatography using miniature columns enabled 1-step purification of recombinant antibodies from clarified cell culture fluid (CCCF) (Repligen/Cytiva). Size exclusion chromatography (SEC) was performed on Protein A purified material using an AKTA Express system with a 120mL Superdex 16/600 200pg S200 gel filtration attached if additional polishing step were required. The resulting purity by SEC was >90% monomer. Separation of monomeric fractions from high molecular weight aggregates was achieved by pooling fractions of high purity based on analysis of resulting FPLC chromatograms.

### Preliminary Analytical Characterization Assessment

We used a selected subset of analytical characterization assays to evaluate each of the mAbs to ensure they were of reasonable quality. The assays include; size-exclusion chromatography UPLC, hydrophobic chromatography, reverse phase chromatography, T_onset_/T_m_/T_agg_ measurements by nano-DSF, and sodium dodecyl sulfate capillary electrophoresis. Results of these assays may be found in **Supplementary Table 1** and detailed protocols and methods for each of these can be found in our recent publication (35). The most consistent trend between parental and mutant IgGs is that the T_m_ onset was lower in all RG mutants than the parental counterpart. Characterization of the mAbs suggested they were within acceptable ranges for our studies.

### Antibody Apparent Affinity to Spike Protein *via* Biacore

Antibody binding experiments were performed on a Biacore 4000 instrument using a Streptavidin chip (Cytiva) at 25° C. The running buffer used was filtered HBS-EP (10 mM Hepes,150 mM NaCl, 3.4 mM EDTA, 0.05% polysorbate 20, pH7.4). Biotinylated CoV-2 spike protein (25nM) was captured on streptavidin surface for 2 minutes. A series of five antibody concentrations serially diluted 3-fold starting from 100nM was prepared in the running buffer and injected for 3 minutes at 30 mL/min followed by 10 minutes of dissociation. Chip surface was regenerated after each injection with a single 30 second pulse of 10 mM glycine–HCl (pH 1.9) at the flow rate of 10 mL/min.

### Antibody Binding With ELISA

Half area 96-well plates were coated with either CoV-1 or CoV-2 full length spike protein at 1 µg/mL in PBS buffer (25 µL/well) and incubated at 4°C overnight. Plates were then washed three times with PBST (PBS + 0.05% Tween 20) and blocked with blocking buffer (PBS with 5% FBS, 25 ml/well) for 30 minutes at room temperature. Antibodies were diluted in blocking buffer, starting from 10ug/ml and titrated down to 1:5, and added to the 96-well plates. After 60 minutes of incubation at room temperature, plates were washed three times with PBST and anti-human HRP (Jackson ImmunoResearch, 1:4000 diluted in blocking buffer, 25 ml/well) was added to the plates. After 60 minutes of incubation at room temperature, plates were washed 5 times with PBST and TMB developing reagent was added for colorimetric reaction for 2-3 minutes. The reactions were stopped with 0.16 M sulfuric acid and plates were read by plate reader at OD 450 nm – 650 nm.

### Generation of Recombinant VSVΔG-Based Pseudoviruses Carrying Firefly Luciferase Reporter Gene and Coronavirus Spike Proteins

Pseudovirus particles were made as previously described by Whitt (36) and Schmidt et al. (37). Briefly, to generate rVSVΔG-Luc pseudoviruses, 293T cells were seeded at 7 x 10^6^ cells/plate in 10 cm dishes. The next day, the cells were transfected with 12.5 μg pCAGGS plasmids encoding coronavirus spike proteins (CoV-2-S Δ18 or SARS-SΔ19). The following day, transfected cells were infected with rVSVΔG-Luc/VSV-G seed virus (Kerafast.com) at MOI=1. After 24 hrs, supernatant was collected and centrifuged at 1320g 10 min. Supernatant was aliquoted into single-use vials and stored at -80°C.

### rVSVΔG-Luc Pseudovirus Neutralization Assay

Pseudovirus neutralization assay was performed as previously described in Schmidt et al. (37). Briefly, the day before infection, 293T ACE2 cells (Integral Molecular) were seeded in solid white TC-treated 96-well assay plates at 45,000 cells/well. Before infecting target cells, viral stocks were incubated with anti-VSV-G hybridoma I1 (ATCC CRL-2700) supernatant at 10% final concentration for 1 hr at 37°C to neutralize any residual contaminating rVSVΔG-Luc/VSV-G seed virus particles. Meanwhile, 40 μg/mL dilutions of each Ab were prepared in PBS. The samples were then five-fold serially diluted in PBS in triplicate to yield 8 serial dilutions in total per construct. Virus stocks were diluted accordingly in DMEM + 0.35% BSA with an aim to yield ~5 x 10^5^ - 1 x 10^6^ RLU per 20 μL virus when measured using One-Glo Luciferase Assay kit (Promega) the following day in infected cells. 30 μL diluted virus was then added to 96-well U-bottom plate and combined with 30 μL diluted Ab to give final starting Ab concentration of 20 μg/mL. Plates were gently swirled and centrifuged at 1000 rpm for 2 min before incubating for 1 hr at 37°C. After incubation, cell media was gently removed from previously seeded 293T ACE2 cells and 40 μL of virus + Ab mixture was added. Virus infected cells were incubated for 1 hr at 37°C and then 60 μL culture media was added to each well. The infected cells were then cultured overnight. The second day, luciferase assay was performed with One-Glo Luciferase Assay kit (Promega) per manufacturer’s instructions. Briefly, 100 μL luciferase reagent was added to each well and plates were incubated on shaker for 5 min at RT. Luciferase activity was measured using an EnVision Multimode plate reader (Perkin Elmer). The half maximal inhibitory concentration (IC_50_) was calculated in GraphPad Prism using 4-parameter nonlinear regression fit to log relative light units (log RLU).

### Stable Cell Line Generation

CHO-KI suspension cell lines stably expressing SARS-CoV-1 or SARS-CoV-2 spike were generated using aa 1-1236 and aa 1-1254 sequences from the SARS-CoV-1 and SARS-CoV-2 spikes respectively. Cells were cultured in a shaking incubator at 37C, 6% CO2, 75% humidity, and 120RPM. Cells were cultured in a shaking incubator at 37°C, 6% CO_2_, 75% humidity, and 120 RPM. The cells were grown in CD CHO media (Gibco, Cat. 12490) with 4 mM L-glutamine (Avantor, Cat. 2078), 1% Hypoxanthine/Thymidine mix (Gibco Cat. 11067), 4 mg/L Blasticidin (Gibco, Cat. A11139) and 200 mg/L zeocin (Invitrogen, Cat. R25005).

### C1q Binding Assay

C1q binding was assessed using a flow cytometry-based assay modified from Pawluczkowycz et al. (38). CHO-K1 cells stably expressing either the SARS-CoV-1 or SARS-CoV-2 spike protein were used in the experiment. Cells were washed with PBS and stained with fixable viability dye (eBioScience Cat. 65-0866-014) on ice in the dark for 15 mins. Following wash with PBS, 300,000 cells were stained with appropriate antibodies after serial dilution. Purified human C1q (Complement Technology Cat. A099) at 3.5 mg/mL or 35 mg/mL was added to the mixture and incubated at 37°C for 1 h. Cells were washed twice with buffer (BD Cat. 554657) and resuspended in 10% heat-inactivated rabbit serum (Gibco Cat. 16120-099) and incubated on ice for 15 minutes to block non-specific binding. Cells were then incubated with FITC-conjugated rabbit anti-human C1q antibody (Dako, Cat. F0254, 1:100 dilution) at RT for 25 minutes. Cells were washed twice with buffer (BD Cat. 554657) and 10,000 events were detected using Cytoflex flow cytometer (Beckman). GraphPad Prism software was used to plot four-parameter dose-response curves and obtain EC50 values.

### Growth of Live SARS-CoV-1 and SARS-CoV-2

All work with authentic SARS-CoV and SARS-CoV-2 viruses were completed in BSL-3 laboratories at United States Army Medical Research Institute of Infectious Diseases (USAMRIID) in accordance with federal and institutional biosafety standards and regulations. Vero E6 cells were inoculated with either SARS-CoV/Urbani or SARS-CoV-2 (GenBank MT020880.1) at an MOI = 0.01 and incubated at 37°C with 5% CO_2_ and 80% humidity. At 50 hours post-infection, cells were frozen at -80°C for 1 hour, allowed to thaw at room temperature, and supernatants were collected and clarified by centrifugation at ~2500xg for 10 minutes. Clarified supernatant was aliquoted and stored at -80°C. Sequencing data was not completed for the SARS-CoV-1 stock. Sequencing data from SARS-CoV-2 virus stock indicated a single mutation in the spike glycoprotein (H655Y) relative to Washington state isolate MT020880.1.

### Live SARS-CoV-1 and SARS-CoV-2 IFA Neutralization Assay

Authentic SARS-CoV/Urbani, and SARS-CoV-2 at a multiplicity of infection of 0.2, was incubated for 1 h at 37°C with serial dilutions of monoclonal antibodies. Vero-E6 monolayers were inoculated with the antibody-virus mixture at 37°C for 1 hour. Following incubation, viral inoculum was removed and fresh cell culture media was added for an additional 23 hours at 37°C. Cells were washed with PBS, fixed in 10% formalin, permeabilized with 0.2% Triton-X for 10 minutes, and treated with blocking solution. Detection of infected cells was accomplished using an anti-SARS-CoV-1 or anti-SARS-CoV-2 nucleocapsid protein (Sino Biological) detection antibodies, and a goat α-rabbit secondary antibody conjugated to AlexaFluor488. Infected cells were determined using the Operetta high content imaging instrument and data analysis was performed using the Harmony software (Perkin Elmer).

## Results

### Epitope Selection

Introduction of the hexamer-enhancing mutations (HC E345R and E430G), herein referred to as RG mutant, does not guarantee that the selected IgG1 antibody will be able to effectively form a hexamer upon target engagement. This is thought to be due to several factors including binding affinity requirements, dependence on target density and distribution, as well as intrinsic features of the antibody (39).

In hopes of finding antibody molecules that form functional hexamers upon binding to the SARS-CoV-1 and/or SARS-CoV-2 spike proteins, we reviewed the available literature and identified a panel of seven RBD-targeting antibodies with diverse binding affinities, unique epitopes, and differing binding orientations relative to the spike protein (**Figure S1A** & **Table 1**). The selected antibodies represent a wide spectrum of activity profiles against emergent coronaviruses. They are active against either SARS-CoV-1 only, SARS-CoV-2 only, or both SARS-CoV-1 and SARS-CoV-2 in virus neutralization assays (**Table 1**). The panel represents not only a variety of epitopes both in terms of the epitope’s location on the spike protein, but also availability of those epitopes as the RBD domain cycles through the open and closed state (**Table 1**; Epitope Exposure, **Figure S1A**). The spike RBD is known to be highly dynamic. In the closed state, the RBD is positioned close to the core of the spike with the region of the RBD that engages with the host cell receptor hidden from the surface of the protein (**SI Figure 1**) (10, 11). In the open state, the RBD flips out from the spike protein to expose the surface that can then bind to ACE2 (**SI Figure 1**) (10, 11). The selected antibodies represent binders in Class 1, 3, and 4 respectively as designated by Barnes et al. (41). Finally, a NTD targeting antibody (4A8) was selected in addition to antibodies binding to RBD epitopes with a non-RBD binding antibody.

**Table 1:** Selected antibodies, epitopes, and apparent binding affinity to SARS-CoV-1 and SARS-CoV-2 spikes by SPR (top row, Biacore) and ELISA (lower row). Epitope exposure was evaluated in the closed RBD form (**Figure S1b**) and the open RBD form (**Figure S1a**). Epitopes able to bind in either the closed or open form were deemed constitutively exposed, whereas epitopes that become sterically hindered in either the open or closed from were deemed to be dynamically exposed.

Parent Construct	Literature Reported Neutralization (Reference)	Binding Domain	Epitope Exposure	CoV-1		CoV-2	
				Parental IgG Biacore KD (nM) ELISA EC50 (ug/mL)	HC-E345R/E430G mutant Biacore KD (nM) ELISA EC50 (ug/mL)	Parental IgG Biacore KD (nM) ELISA EC50 (ug/mL)	HC-E345R/E430G mutant Biacore KD (nM) ELISA EC50 (ug/mL)
S309	SARS CoV-1 & CoV-2 (16)	RBD- Class 3	Constitutively Exposed	<0.20	<0.20	<0.20	<0.20
				0.02	0.04	0.06	0.08
CR3022	SARS CoV-1 (15)	RBD- Class4	Dynamically Exposed	<0.20	<0.20	0.39	0.43
				0.05	0.04	0.24	0.13
S230	SARS CoV-1 (13)	RBD-Class 1/2	Constitutively Exposed	<0.20	<0.20	No Binding	No Binding
				0.02	0.005	No Binding	3.60
EY6A	CoV-2 (17)	RBD- Class4	Dynamically Exposed	22.8	13.6	0.10	0.29
				3.89	3.82	0.7	0.35
REGN10933	CoV-2 (14, 40)	RBD- Class 1	Dynamically Exposed	No Binding	No Binding	<0.20	<0.20
				1.85	No Binding	0.02	0.02
REGN10987	CoV-2 (14, 40)	RBD- Class3	Constitutively Exposed	No Binding	No Binding	<0.20	<0.20
				3.3	No Binding	0.03	0.03
4A8	CoV-2 (18)	NTD	Constitutively Exposed	No Binding	No Binding	2.21	2.24
				No Binding	No Binding	0.02	0.03

Apparent binding affinity of the antibodies to the SARS-CoV-1 and SARS-CoV-2 trimer spike proteins was measured using SPR (**Table 1**). All antibodies were found to have affinities between < 200pM to ~23nM, in agreement with the literature (references in **Table 1**). Apparent binding affinity of the RG mutant IgG1 was roughly equivalent to the parental antibody which is in agreement with previous findings (31). EY6A demonstrated the greatest difference in apparent binding affinity between mutant and the parental IgG1 in both SARS-CoV-1 and SARS-CoV-2. Notably, the relative improvement did not hold across viruses, parental EY6A bound 2.8x more tightly to SARS-CoV-2, while mutant EY6A bound 1.7x more tightly to CoV-1. This finding in combination with the relative equivalence in binding between the RG mutant and parental in all other antibodies suggests that the hexameric mutations do not directly impact apparent binding affinity.

Given that the dissociation rate of many of the mAbs was below the limit of detection of our SPR instrument (< 10^-5^ s^-1^), we further characterized mAb binding using an ELISA-based assay. The ELISA assay provided additional resolution amongst the mAbs tested and the binding trends were similar to those observed by SPR. The largest differences between the mutant and parental IgG1 were observed for S230 and the SARS-COV-1 spike and a similar trend was observed for SARS-COV-2 binding to both CR3022 and EY6A. However, both REGN10987 and REGN10933 parental mAbs resulted in measurable binding to the SARS-CoV-1 spike protein whereas the mutant IgG1 did not. As noted above, the differences in observed binding are thought to be relatively minor and the findings support the assertion that the hexamer-enhancing mutations do not significantly impact apparent binding affinity.

### Epitope Binding Is Necessary, but Not Sufficient for Hexamer Formation

**Figure 1:**
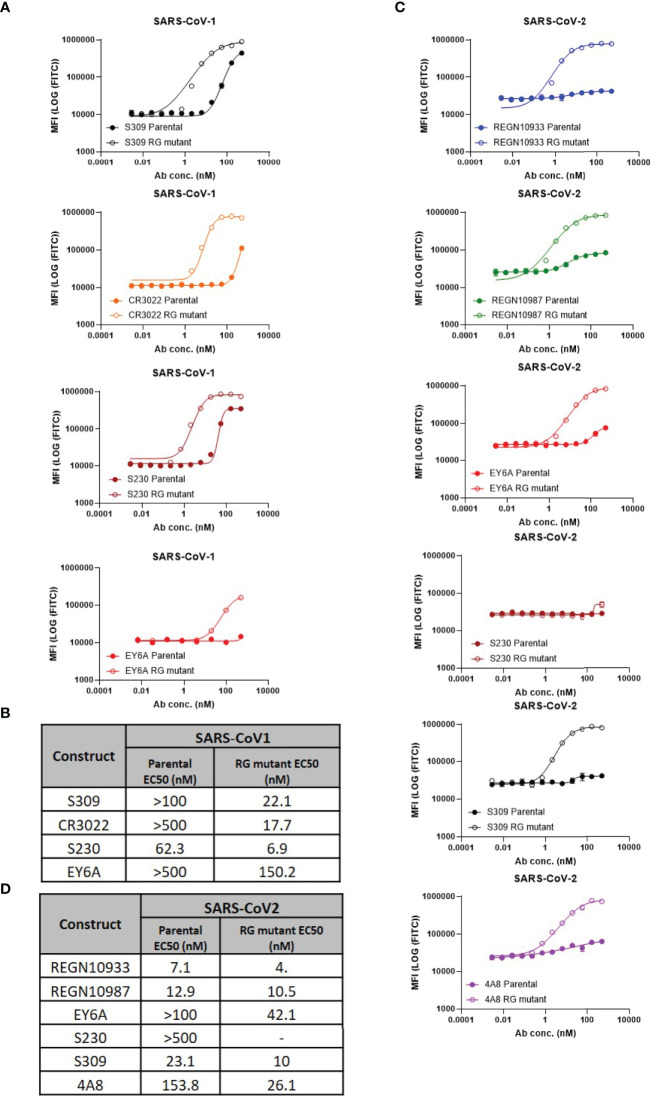
Hexamer formation detected using FACS-based C1q binding assay. **(A)** Comparison of C1q binding of parental vs antibodies with RG mutant mAbs on SARS-CoV-1 cell lines (mean values with SD; n=3; EY6A graphs contain mean values only), **(B)** Calculated EC50 values of RG mutant vs parental, **(C)** Comparison of C1q binding of parental vs antibodies with RG mutants on SARS-CoV-2 cell lines (mean values with SD; n = 3), **(D)** Calculated EC50 values of RG mutant vs parental.

To measure the ability of each antibody-epitope pair to form a hexamer, we employed a modified version of the C1q FACS assay described by Pawluczkowycz et al. (38) (**Figure 1**). C1q is known to bind preferentially to fully formed IgG hexamers (31) and, therefore, can serve as a surrogate measure of hexamer formation. Previous studies have shown that C1q binding is observed in wildtype hIgG1, but not hIgG4, and this corresponds to the ability of those antibodies to drive complement-dependent cytotoxicity (38). In our studies, C1q binding was observed to bind to some, but not all, parental antibodies (e.g, S230 and S309 to CoV1 spike). Introduction of the RG mutation increased C1q binding for all neutralizing antibody:spike pairs (**Figures 1A, C**) For five antibody:spike pairs (S230 RG mutant:CoV-2 spike, REGN10933 RG mutant: CoV1 spike, REGN10987 RG mutant: CoV1 spike, 4A8 RG mutant: CoV1 spike, 4A8 parental: CoV1 spike) where the antibody did not bind to the target spike protein by SPR or ELISA (**Table 1**), C1q binding was also not observed (data not shown).

### Hexamer-Enhanced Antibodies Improve Neutralization in Pseudo- and Live SARS-CoV-1 and SARS-CoV-2 Viruses

Due to the difficulties in assessing the live SARS-CoV-1 & SARS-CoV-2 viruses, namely the requirement of BSL3 biosafety containment, we generated VSV-based pseudoviruses carrying the SARS-CoV-1 or SARS-CoV-2 spike proteins on the surface according to an established method (36). We measured the neutralizing abilities of the above-mentioned antibodies against these VSV pseudoviruses.

**Figure 2:**
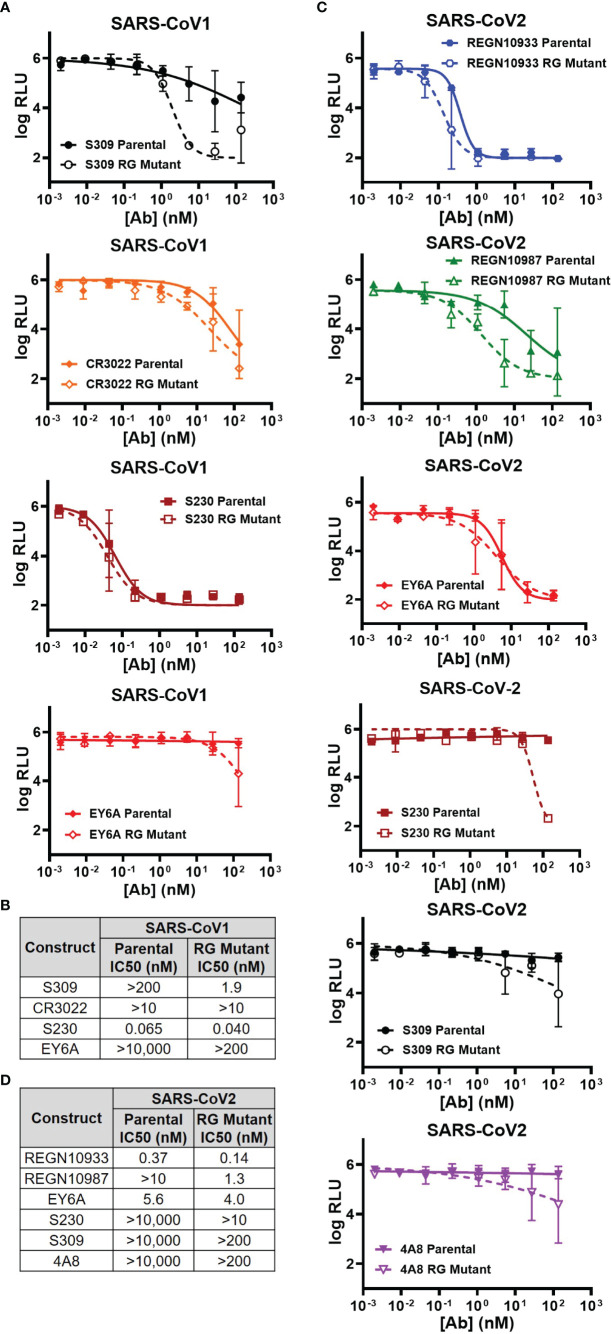
Comparison of the parental and RG mutant antibodies by using rVSVΔG-Luc pseudovirus neutralization assay. **(A)** SARS-CoV1 pseudovirus neutralization by 4 antibodies (S309, CR3022, S230 & EY6A) (mean RLU values with SD; n = 3). **(B)** Calculated IC_50_ neutralization values against CoV1 pseudovirus by RG mutant vs parental. **(C)** SARS-CoV-2 pseudovirus neutralization by 6 antibodies (REGN10933, REGN10987, EY6A, S230, S309 & 4A8) (mean RLU values with SD; n = 3). **(D)** Calculated IC_50_ neutralization values against CoV-2 pseudovirus by RG mutant vs parental. Data for non-neutralizing constructs may be found in the Supplemental information (**SI Figure 2**).

Three antibodies (S309, CR3022, S230) were previously reported to be able to neutralize either live SARS-CoV-1 or its pseudovirus (15, 16, 42). We compared the neutralizing abilities of both parental and RG mutant IgG1 in our VSV pseudovirus neutralization assay (**Figure 2A**). In agreement with published data, all 3 antibodies demonstrated neutralization abilities against SARS-CoV-1 pseudovirus (**Figure 2A**). Surprisingly, although the SPR and ELISA binding affinities to the CoV-1 spike protein are similar between parental and RG mutant IgG1 (**Table 1**), the latter clearly shows improvement in neutralizing ability in our SARS-CoV-1 pseudovirus assay (**Figure 2A**). The degree of improvement for these 3 antibodies can be seen from estimated IC50 values of both the parental and RG mutant forms (**Figure 2B**). Although antibody EY6A only binds but does not neutralize SARS-CoV-1 [**Table 1**, & Ref (17)], its RG mutant gained some neutralizing ability at higher concentrations (**Figures 2A, B**). We also tested 3 non-neutralizing antibodies (REGN10933, REGN10987, 4A8), none of the RG mutants showed improved neutralization for these antibodies (**SI Figure 2A**).

Similarly, we analyzed the neutralizing abilities of 7 antibodies (REGN10933, REGN10987, EY6A, S230, S309, 4A8 and CR3022) against the SARS-CoV-2 pseudovirus (**Figure 2C** & **SI Figure 2B**). The RG mutant forms of the 6 antibodies (REGN10933, REGN10987, EY6A, S230, S309, 4A8) also showed significant improvements in our neutralization measurement when compared with their parental IgGs (**Figure 2C**). We calculated the estimated IC50s for each of the antibodies and clearly the improvement on neutralization by the RG mutants was observed (**Figure 2D**). For CR3022, there is no detectable improvement by the RG form (**SI Figure 2B**)

**Figure 3:**
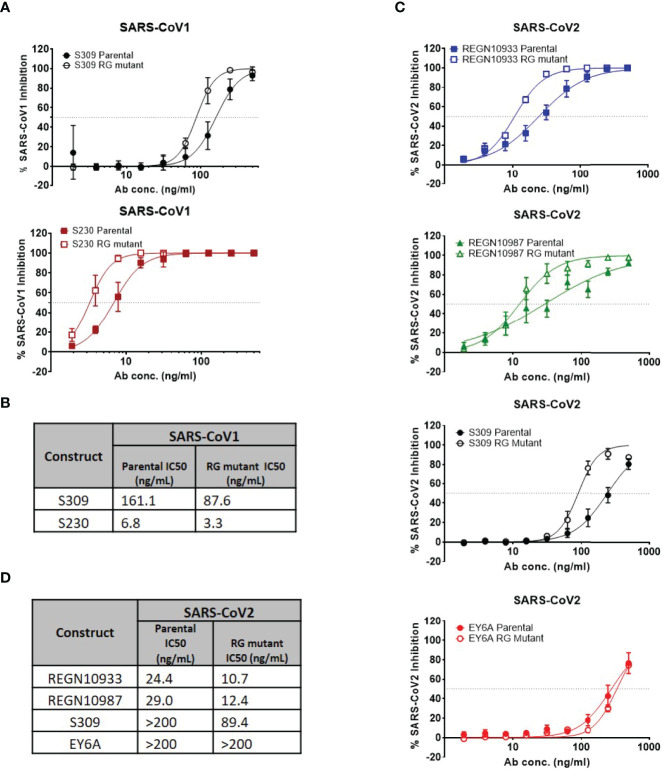
Authentic (live) virus neutralization assays for SARS-CoV-1 and SARS-CoV-2. **(A)** Percent SARS-CoV-1 neutralization with select antibodies (mean values with SEM; n = 4). **(B)** Calculated IC_50_ neutralization values for SARS-CoV-1. **(C)** Percent SARS-CoV-2 neutralization with select antibodies (mean values with SEM; n = 4). **(D)** Calculated IC_50_ neutralization values for SARS-CoV-1. Data for non-neutralizing constructs may be found in the Supplemental information (**SI Figure 3**).

To determine whether enhancement in neutralization potency is observed with live SARS-CoV-1 and SARS-CoV-2 viruses, we assessed neutralization potency using the same set of antibodies in a live virus assay (**Figure 3**). With live SARS-CoV-1 virus, neutralization potency was enhancedin the hexamer-enhanced variants of S309 and S320 based on estimated IC50s (**Figures 3A, B**). Both CR3022 parental and RG mutant antibodies did not show neutralization in this assay (**SI Figure 3**). Likewise, with live SARS-CoV-2 virus, the RG mutations also enhanced neutralization potency of S309, REGN10933 and REGN10987 antibodies (**Figures 3C, D**). While it is difficult to directly compare the degree of changes across the different viral neutralization assays due to inherent differences between the assays, the trends in improvement are consistent. Interestingly, the RG mutant form of EY6A antibody did not show improved neutralization potency in this assay.

## Discussion

Hexamer-enhancing IgG mutants and related multimeric formats are gaining traction throughout the scientific community and numerous examples are emerging in the literature against targets including Trem2 (43), DR-5 (33), CD-38 (44), CD-37 (45), gonococcal LPS (46) and as yet unreleased targets (47). Our *in vitro* neutralization assays using live SARS-CoV1 and SARS-CoV-2 suggest that hexamer-enhancing mutations can improve mAb potency. Our findings are in line with several recent reports that multimeric formats increase anti-viral potency and ultimately lead to improved neutralization (25–28). However, to our knowledge, this is the first time that the use of hexamer-enhanced IgG1 has been disclosed for a viral target and our findings suggest that this may be a potential approach for anti-viral therapeutic antibodies. Furthermore, the trend in neutralization potency was consistent between the pseudovirus and the live virus. Although modest, even a slight improvement in potency could have significant consequences for global supply as manufacturing is seen as a key bottleneck for delivering life-saving SARS-CoV-2 therapies (24).

One of the key biological roles of hexamer formation by IgG1 molecules is the recruitment of C1q and activation of the complement cascade (31). However, the viral neutralization assays reported here were performed in the absence of complement components suggesting that the observed improvement in neutralization is independent of complement. A few possible explanations for the improved neutralization in the absence of C1q may be; 1) improved avidity from multimerization and enhanced Fc-Fc interaction or 2) steric hindrance of the spike protein by the mAb hexamer, effectively forming a physical buffer or cap between the viral spike proteins and the ACE-2 receptor on host cells. In agreement with previous reports, the addition of C1q further improves neutralization in the RG format (**SI Figure 4**) however, further exploration of the hexamer-enhanced format, for example through structural studies, will be necessary to elucidate exact mechanism of neutralization in the absence of C1q.

These findings also call into question whether the inherent capability of hexameric antibodies to activate the complement cascade and induce complement dependent cytotoxicity (CDC) could be leveraged to further improve the neutralization capacity of hexamer-enhanced mAbs in the context of viral infection. It is well documented that activation of the complement cascade on viral surfaces through the lectin pathway can contribute to either a protective or pathogenic outcome depending on the context and type of viral infection (48). In the case of SARS-CoV-2 infection, the role of complement is still not entirely understood. Overactivation of complement has been associated with increased mortality (49), but many questions remain around the importance of how the complement system is activated (50), what epitopes are involved (51), and the degree of complement activation (52). Encouragingly, literature reports suggest that complement activation and CDC induced by hexamer formation may be tunable to some degree within the hexamer-enhancing format, opening the possibility to match viral biology to CDC-enhancing properties of a specific mAb (53). These mutations include P329R/A which are thought to inhibit C1q binding, but also mutations at other sites including K322, E269, N297 and several others (33, 53).

In addition to a better understanding of the role of complement in SARS-CoV2, clinical application of hexamer-enhanced IgGs for anti-viral use would require further optimization of the mutation sites and selections as well as extensive additional *in-vitro* and *in-vivo* studies. Importantly the relationship drug clearance and viral neutralization would need to be explored as there is a known propensity for some hexamer enhancing mutants to bind compliment in solution (34). Whether hexamer-enhanced mAbs would improve viral neutralization *in-vivo* is yet to be determined.

While there are still several open questions around the use of hexamer-enhancing mutations in anti-viral applications, the format itself shows significant promise. Encouragingly, two hexamer-enhanced IgG molecules have already entered the clinic (NCT03576131, NCT04824794) for oncological indications with more likely to follow.

## Data Availability Statement

The original contributions presented in the study are included in the article/**Supplementary Material**. Further inquiries can be directed to the corresponding author.

## Author Contributions

KP, SH, and LK designed the research. MS, QG, SS, AS, KV, XM, TB, S-JC, FR, DGrandy, KS, RT performed the experiments. KP, SH, DGorman, LZ, AC, LF-D, and LK performed analysis. All authors contributed to writing and review of the paper.

## Funding

This study was funded by Merck & Co., Inc. The funder was not involved in the study design, collection, analysis, interpretation of data, the writing of this article or the decision to submit it for publication.

## Conflict of Interest

All authors are employees of Merck Sharp & Dohme Corp., a subsidiary of Merck & Co., Inc., Kenilworth, NJ, USA.

## Publisher’s Note

All claims expressed in this article are solely those of the authors and do not necessarily represent those of their affiliated organizations, or those of the publisher, the editors and the reviewers. Any product that may be evaluated in this article, or claim that may be made by its manufacturer, is not guaranteed or endorsed by the publisher.
