# Supplementary material for: Hexamerization of Anti-SARS CoV IgG1 Antibodies Improves Neutralization Capacity
Source: Front Immunol. 2022 May 4;13:864775. doi: 10.3389/fimmu.2022.864775 (PMC9114490; doi:10.3389/fimmu.2022.864775)
Supplement: Supplementary file 1 [file DataSheet_1.docx]

Supplementary Material

**Hexamerization of anti-SARS CoV IgG1 antibodies improves neutralization capacity**

**Kalyan Pande^1†^, Scott A. Hollingsworth^2†^, Miranda Sam^1^, Qinshan Gao^1^, Sujata Singh^1^, Anasuya Saha^1^, Karin Vroom^1^, Xiaohong Shirley Ma^1^, Tres Brazell^1^, Dan Gorman^1^, Shi-Juan Chen^1^, Fahimeh Raoufi^1^, Marc Bailly^1^, David Grandy^1^, Karthik Sathiyamoorthy^1^, Lan Zhang^3^, Rob Thompson^2^, Alan C. Cheng^2^, Laurence Fayadat-Dilman^1^, Bernhard H. Geierstanger^1^, Laura J. Kingsley^1^***

^1^ Discovery Biologics, Merck & Co., Inc., 213 East Grand Ave., South San Francisco California 94080, USA

^2^ Discovery Chemistry, Merck & Co., Inc., 213 East Grand Ave., South San Francisco California 94080, USA

^3^ Infectious Disease and Vaccine Discovery, Merck & Co., Inc., 770 Sumneytown Pike, West Point, PA 19486, USA

^†^denotes equal contribution

*** Correspondence:**Corresponding Author
[Laura.kingsley@merck.com](mailto:Laura.kingsley@merck.com), Merck & Co., Inc., 213 East Grand Ave. South San Francisco California 94080, USA; ORCID: 0000-0002-5566-3974

**Keywords: hexameric antibody, SARS-CoV, viral neutralization, thermostability**


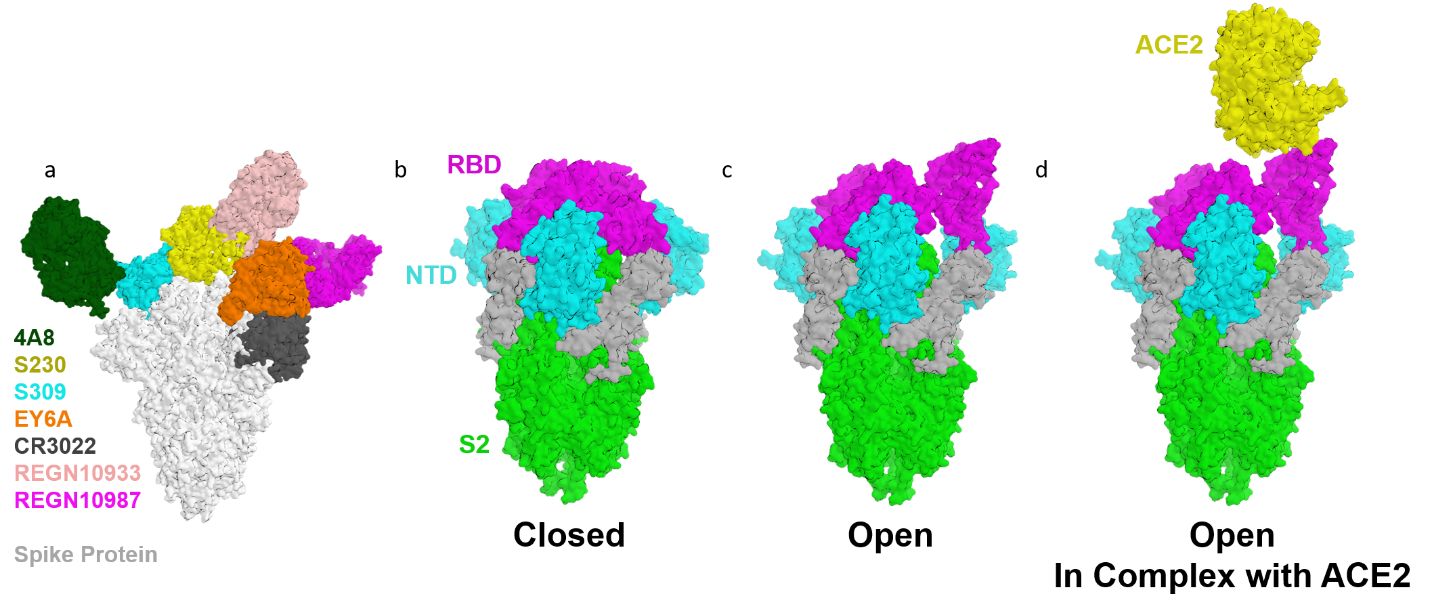


**SI Figure 1.** a) Selected antibody binding sites. The spike is shown in light grey and the selected panel of antibodies in various colors to demonstrate the different epitopes and binding orientations for our selections. Conformational states of the spike protein and RBD. **b**) closed conformation (PDB ID 6VXX), **c**) single RBD in the open/up conformation (PDB ID 6YVB), **d**) open conformation bound the human ACE2 receptor (PDB ID 7A91).

**
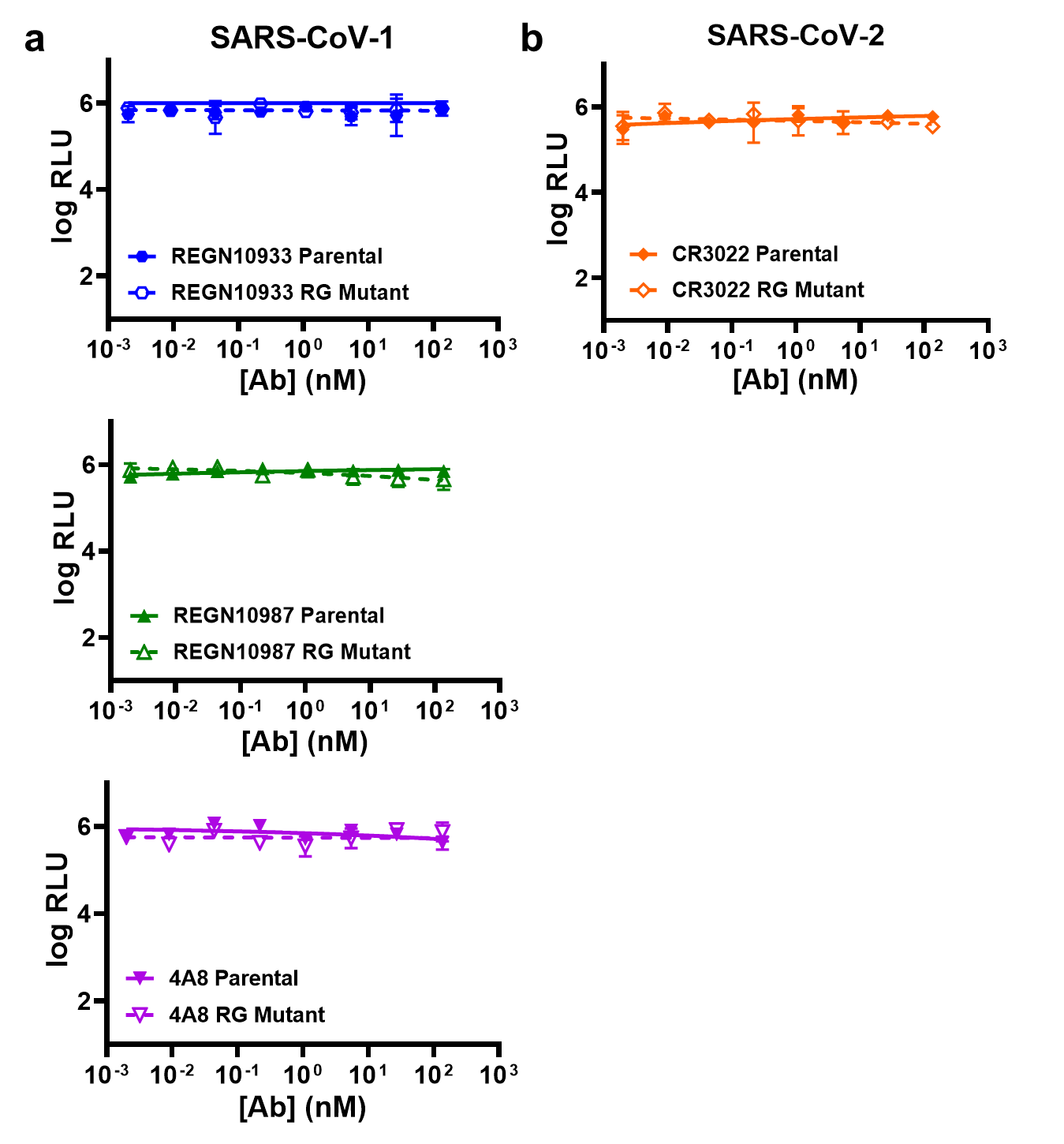
**

**SI Figure 2**. Comparing the parental and RG mutant forms of non-neutralizing antibodies by using rVSVΔG-Luc pseudovirus neutralization assay. a) SARS-CoV1 pseudovirus neutralization by 3 antibodies (REGN10933, REGN10987, 4A8) (mean RLU values with SD; n=3). b) SARS-CoV2 pseudovirus neutralization by CR3022 mean RLU values with SD; n=3).

**
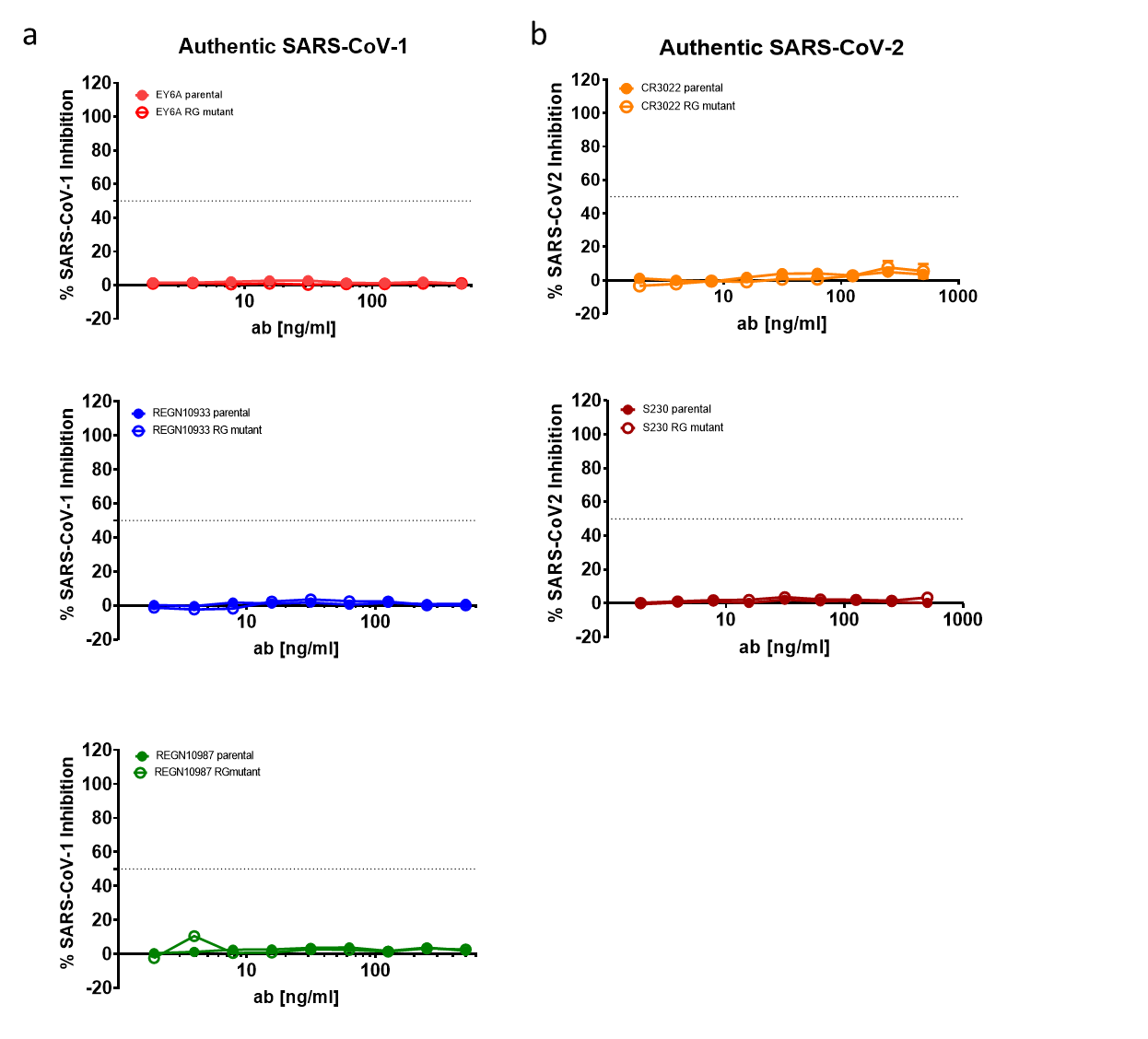
**

**SI Figure 3.** Live neutralization results for non-neutralizing mAbs in **a)** SARS-CoV-1 and **b)** SARS-CoV-2


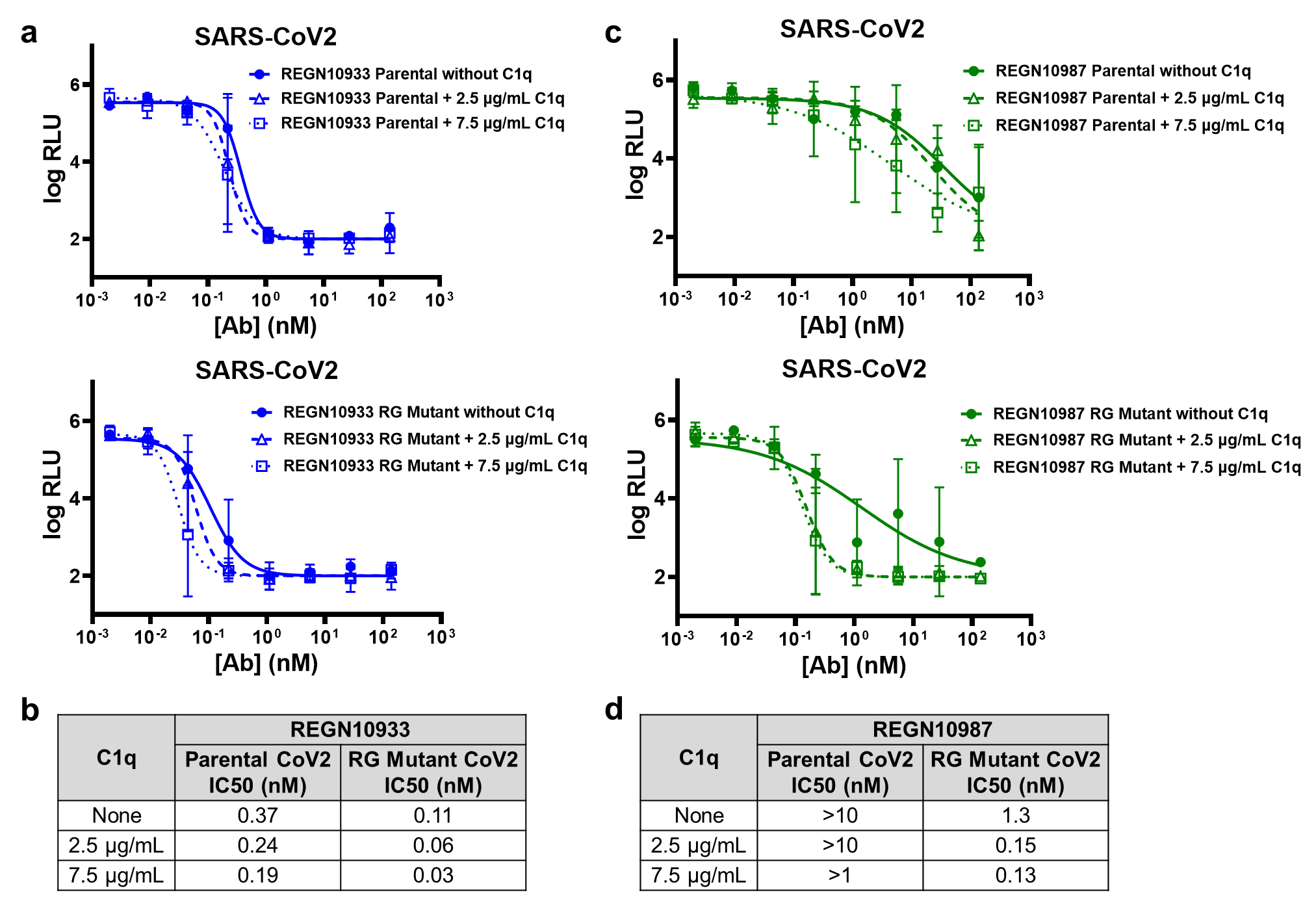


**SI Figure 4.** Addition of C1q to rVSVΔG-Luc pseudovirus neutralization assay with REGN constructs. Assay was performed as in Method 2.7 except with addition of purified C1q (Complement Technology) to virus + Ab mixtures where applicable. a) SARS-CoV2 pseudovirus neutralization by REGN10933 parental and RG mutant without C1q, with 2.5 μg/mL C1q, and with 7.5 μg/mL C1q (mean RLU values with SD; n=3). b) Calculated IC_50_ neutralization values against CoV2 pseudovirus by REGN10933 parental and RG mutant. c) SARS-CoV2 pseudovirus neutralization by REGN10987 parental and RG mutant without C1q, with 2.5 μg/mL C1q, and with 7.5 μg/mL C1q (mean RLU values with SD; n=3). d) Calculated IC_50_ neutralization values against CoV2 pseudovirus by REGN10987 parental and RG mutant.

| **Mutation** | **Titer** | **UPSEC** | | | | **RPHPLC** | | | | **CE-SDS NR** | | | **CESDS Red** | | | **HIC** | | | | **NanoDSF** | | | | |
| --- | --- | --- | --- | --- | --- | --- | --- | --- | --- | --- | --- | --- | --- | --- | --- | --- | --- | --- | --- | --- | --- | --- | --- | --- |
|  | Yield  (mg/L) | HMW | Main Peak | LMW | RT (min) | Pre- Peak | Main Peak | Post Peak | RT (min) | LMW | Main Peak | HMW | Lc | Hc | Other Species | Pre- Peak | Main | Post Peak | RT (min) | Tm Onset | Tm_1_ | Tm_2_ | Tm_3_ | T_agg_ |
| 48A | 13.2 | 2.11 | 97.90 | - | 2.38 | 1.59 | 98.4 | - | 2.21 | 3.28 | 96.70 | - | 36.2 | 63.4 | 0.40 | - | 100.0 | - | 16.97 | 49.6 | 58.5 | 68.8 | 81.3 | 59.6 |
| 48A RG mutant | 42.3 | 20.72 | 79.30 | - | 2.36 | 10.63 | 89.4 | - | 2.20 | 7.31 | 92.70 | - | 35.9 | 63.8 | 0.31 | 36.28 | 63.7 | - | 17.10 | 48.2 | 57.8 | 66.7 | 77.2 | 57.6 |
| REGN10987 | 13.0 | 3.16 | 96.90 | - | 2.48 | 4.58 | 95.4 | - | 2.49 | 4.00 | 96.00 | - | 23.1 | 74.9 | 1.94 | - | 100.0 | - | 26.52 | 62.4 | 74 | - | - | 75.6 |
| REGN10987 RG mutant | 112.3 | 4.46 | 95.50 | - | 2.47 | 11.21 | 88.8 | - | 2.48 | 7.49 | 92.50 | - | 25.5 | 73.3 | 1.20 | - | 100.0 | - | 26.63 | 51.5 | 60.4 | 74.5 | - | 72.2 |
| CR3022 | 13.7 | 0.79 | 99.20 | - | 2.51 | 0.95 | 98.5 | 0.53 | 2.52 | 1.27 | 98.70 | - | 26.0 | 74.0 | 0.00 | - | 100.0 | - | 23.32 | 56.4 | 70.9 | 77.2 | - | 78.1 |
| CR3022 RG mutant | 46.3 | 17.18 | 45.40 | 37.40 | 2.38 | 2.26 | 96.6 | 1.17 | 2.52 | 3.00 | 97.00 | - | 29.0 | 70.7 | 0.36 | - | 69.4 | 30.7 | 23.39 | 52.2 | 60.8 | 76.4 | - | 76.1 |
| S230 | 90.0 | 0.75 | 99.30 | - | 2.55 | 0.95 | 98.6 | 0.47 | 2.16 | 2.79 | 97.20 | - | 28.8 | 71.0 | 0.13 | - | 100.0 | - | 20.23 | 61.7 | 68 | 82 | - | 67.1 |
| S230 RG mutant | 17.0 | 2.30 | 97.70 | - | 2.53 | 0.96 | 96.6 | 2.47 | 2.18 | 2.11 | 97.90 | - | 30.5 | 69.4 | 0.09 | - | 100.0 | - | 20.42 | 50.1 | 66.2 | - | - | 63.7 |
| REGN10933 | 50.7 | 1.36 | 98.60 | - | 2.45 | 0.98 | 99.0 | - | 2.46 | 1.84 | 98.20 | - | 25.4 | 74.2 | 0.37 | 22.87 | 77.1 | - | 18.65 | 63.3 | 68.8 | 82 | - | 69.1 |
| REGN10933 RG mutant | 22.0 | 3.27 | 96.70 | - | 2.44 | 2.09 | 97.9 | - | 2.45 | 2.72 | 97.30 | - | 30.0 | 69.4 | 0.57 | 40.66 | 59.3 | - | 18.76 | 56.7 | 67.5 | - | - | 66.5 |
| S309 | 89.3 | 1.51 | 95.51 | 2.99 | 3.36 | 0.55 | 99.5 | - | 2.17 | 1.69 | 98.30 | - | 28.9 | 71.0 | 0.14 | - | - | 100.0 | >40 | 69.1 | 70.7 | 82 | - | 69.8 |
| S309 RG mutant | 14.6 | 30.11 | 69.89 | - | 3.35 | 13.91 | 78.2 | 7.92 | 2.19 | 16.16 | 83.84 | - | 36.1 | 62.8 | 1.12 | - | - | 100.0 | >40 | 55.9 | 60 | 70 | 77.9 | 69.3 |
| EY6A | 35.0 | 0.32 | 99.68 | - | 2.58 | - | 100.0 | - | 2.30 | 2.18 | 97.82 | - | 29.3 | 70.5 | 0.19 | - | - | 100.0 | >40 | 62.2 | 76.4 |  |  | 76.6 |
| EY6A RG mutant | 4.0 | 1.83 | 44.88 | 42.11 | 2.55 | 6.37 | 93.7 | - | 2.29 | 5.79 | 94.21 | - | 27.8 | 71.8 | 0.43 | - | - | 100.0 | >40 | 53.9 | 60.9 | 76.5 | - | 74.7 |

**Supplemental Table 1.** Biophysical characterization of mAb. The most consistent trend between parental and mutant IgGs is that the Tm onset was lower in all RG mutants than the parental counterpart. On average, RG mutant antibodies had a Tm onset that was 8°C lower than the parental mAb
